# Rheological Behavior of Polymer/Carbon Nanotube Composites: An Overview

**DOI:** 10.3390/ma13122771

**Published:** 2020-06-18

**Authors:** Rossella Arrigo, Giulio Malucelli

**Affiliations:** Department of Applied Science and Technology, and Local INSTM Unit, Politecnico di Torino, Viale Teresa Michel 5, 15121 Alessandria, Italy; giulio.malucelli@polito.it

**Keywords:** polymer nanocomposites, carbon nanotubes (CNTs), rheological behavior, viscoelastic properties, rheological percolation

## Abstract

This paper reviews the current achievements regarding the rheological behavior of polymer-based nanocomposites containing carbon nanotubes (CNTs). These systems have been the subject of a very large number of scientific investigations in the last decades, due to the outstanding characteristics of CNTs that have allowed the formulation of nanostructured polymer-based materials with superior properties. However, the exploitation of the theoretical nanocomposite properties is strictly dependent on the complete dispersion of CNTs within the host matrix and on the consequent development of a huge interfacial region. In this context, a deep knowledge of the rheological behavior of CNT-containing systems is of fundamental importance, since the evaluation of the material’s viscoelastic properties allows the gaining of fundamental information as far as the microstructure of nanofilled polymers is concerned. More specifically, the understanding of the rheological response of polymer/CNT nanocomposites reveals important details about the characteristics of the interface and the extent of interaction between the two components, hence allowing the optimization of the final properties in the resulting nanocomposites. As the literature contains plenty of reviews concerning the rheological behavior of polymer/CNT nanocomposites, this review paper will summarize the most significant thermoplastic matrices in terms of availability and relevant industrial applications.

## 1. Introduction

Over the last decades, polymer-based nanocomposites have attracted tremendous research interest, as the addition of very low nanofiller loadings allows the obtaining of a remarkable enhancement of the final properties of a large number of polymeric systems, determining new perspectives for the continuous demand of advanced materials [1,2]. In particular, polymer nanocomposites exhibit improved structural and functional properties as compared to their corresponding unfilled counterparts, while maintaining the versatility and feasibility of polymer processing and manufacturing [3]. Therefore, a number of innovative materials with tailorable mechanical, optical or electrical properties, potentially suitable for several high-tech applications (concerning the biomedical, electronic or automotive fields, among a few), has been designed and produced [4,5]. In this context, a huge variety of nanofillers, differing in terms of chemical nature, shape and morphology, has been assessed and then exploited; more specifically, several kinds of nanoparticles, including metals [6], ceramics [7] or carbon-based [8] fillers, characterized by two-dimensional (platelets) [9], one-dimensional (rod-like) [10] or zero-dimensional (sphere) [11] shapes, have been embedded in numerous thermoplastic or thermoset matrices, with the aim of obtaining high-performing materials, able to continuously widen the polymer markets.

Among the aforementioned types of nanofillers used in polymer-based nanocomposites, carbon nanotubes have demonstrated great potential for the development of new nanostructured materials [12,13]. Since their discovery, carbon nanotubes (CNTs) have been the focus of considerable research effort, due to their outstanding physical and chemical properties [14,15]. In particular, CNTs possess unique electronic properties [16], high thermal conductivity [17] and exceptional mechanical properties [18] that, coupled with their low density, offer the opportunity to develop innovative polymer-based nanocomposites.

CNTs are rod-like particles, constituted of covalently bonded carbon atoms arranged in a hexagonal lattice. Besides, CNTs may exist as single-walled tubes, formed by the rolling up of a single graphene layer, or multi-walled tubes, consisting of several rolled graphene layers, coaxially arranged around a central hollow core [19]. Single-walled carbon nanotubes (SWCNTs) present an electrically conductive or semi-conductive character, depending on the chirality of the graphene lattice, while multi-walled carbon nanotubes (MWCNTs) are conductors [20]. In general, more complex production methods and purification procedures are required for the obtainment of regular and not defective SWCNTs, whose utilization is usually limited to niche applications, especially in the electronics field; on the other hand, more industrially oriented production strategies for MWCNTs allow their exploitation as fillers in several polymer-based composite materials with commercial applications.

However, the potentialities of CNTs in providing enhanced properties when embedded in polymers are strongly dependent on their state of dispersion within the host matrix; in fact, CNTs are prone to aggregate when embedded in a viscous medium, and this issue considerably limits the full exploitation of their theoretical excellent properties [21,22]. Therefore, with the aim of achieving a uniform dispersion of CNTs and to control the material microstructure, a large number of processing methods has been designed and exploited, including either traditional approaches (i.e., solution mixing or melt-mixing) [23] or innovative strategies (such as the Layer-by-Layer method) [24]. Besides, a further strategy to improve the dispersion of CNTs within host matrices and to enhance polymer/filler compatibility relies on the modification of their outer surface through the immobilization, both covalent and non-covalent, of different functional groups, able to promote the establishment of strong interactions in the interfacial region [25,26].

Apart from the level of CNT dispersion, a further key factor for achieving nanocomposite systems endowed with superior properties is the control of the interfacial region and of the established polymer/nanofiller interactions [27]. In a polymer-based multiphase system, the macromolecules located within the interfacial region exhibit some peculiar characteristics with respect to those in the bulk; more specifically, in close proximity to the nanofiller surface, the polymer chains are in a perturbed state and their configuration depends on the strength of the interactions taking place between the filler and the matrix [28].

Therefore, to fully unlock the potential of CNTs for applications in polymer-based nanocomposites, a comprehensive understanding of the characteristics of the interface and of the mechanism of the formation of the matrix/nanofiller interactions is essential.

A very powerful tool for assessing the state of dispersion of the nanofiller in a polymeric matrix, as well as the filler–polymer interactions established at the interface, is the evaluation of the material’s rheological behavior. In fact, the rheological functions of polymer-based complex systems reveal fundamental information about the microstructure of nanofilled polymers, allowing an accurate characterization of the nanocomposite’s internal structure, as well as a full understanding of the possible evolution of the material’s structure [29,30]. In particular, the assessment of the rheological response of polymer nanocomposites allows the gaining of a fundamental insight into the characteristics of the interfacial region, as the perturbed state of the polymer chains interacting with nanofillers involves a modification of the macromolecules’ dynamics, with a consequent variation of the polymer relaxation spectrum [31]. Furthermore, rheological measurements are very useful for gaining a fundamental insight into the organization of the embedded nanoparticles into the host matrix and their possible arrangement in complex architectures. In fact, when the embedded nanoparticles are well dispersed and the formation of a continuous path of nanoparticles spanning the matrix occurs, the motion of long polymer chain segments is restricted, thus resulting in a dramatic change in the relaxation dynamics of the polymer macromolecules [32,33]. The formation of the aforementioned nanofiller network throughout the host polymer indicates that the rheological percolation threshold has been reached; as a consequence, the viscoelastic behavior of the polymer matrix is significantly affected [34]. The nanofiller content corresponding to the percolation threshold depends on the morphology of the nanofiller and on its homogeneity of dispersion: in particular, using nanofillers with high aspect ratios or improved dispersion allows the reaching of the percolation transition at low concentrations [35]. More specifically, the introduction of fillers with high aspect ratios, such as CNTs, allows the reaching of the percolation threshold at a lower filler content as compared to other carbon-based particles with different geometrical characteristics [36]. As an example, in the case of carbon black (CB)-containing polymer composites, the content of CB needed to form a percolative pathway through the matrix is typically about 10 wt.%, whereas this content is remarkably reduced by using CNTs or graphene-like fillers [37,38]. However, irrespectively of the particle geometrical characteristics, the loading of fillers, at which the formation of the percolation network occurs, is strictly related to the state of dispersion of the particles within the host matrix, which represents a crucial factor controlling the arrangement of embedded fillers in complex structures.

Usually, the formation of a rheological percolative network is characterized through dynamic oscillatory measurements performed at low frequencies, which reveal a deviation of the material’s rheological behavior from the classical theory of linear viscoelasticity [39]. In particular, a transition from a liquid-like to solid-like rheological response is observed, ascribed to the disappearance of the Newtonian plateau in the complex viscosity trend and the flattening of the storage and loss moduli curves in the low frequency region [40,41].

As regards polymer nanocomposites, the addition of carbon nanotubes may affect the matrix rheological behavior in different ways, depending on their loading, aspect ratio and possible orientation within the host matrix [42,43]. Generally, the rheological behavior of unaligned CNTs dispersed into a polymer matrix can be classified into three regimes [44]. First, at low nanofiller loadings, CNTs are dispersed as individual entities and only short-range intertube interactions are possible: this scenario is called the dilute regime. As the nanofiller content increases, the formation of a percolation network occurs and a transition from the dilute to the semi-dilute regime is observed; in this regime, the rheological behavior of the nanocomposite is governed by the filler/filler interactions. For CNT contents higher than the percolation threshold, the concentrated regime is reached and the rheological functions tend to approach asymptotic values.

In this review, the rheological behavior of CNT-embedded polymer nanocomposites is thoroughly discussed. In particular, the general features of the viscoelastic response of these systems are summarized, considering the possible influence of the processing method selected for the nanocomposite preparation. Since the investigation of the viscoelastic properties of polymer/CNT nanocomposites has been the subject of a huge number of scientific reports, the discussion will be focused on the most significant matrices in terms of availability and relevant applications; in addition, a brief section will be devoted to the rheological behavior of CNT-containing polymer blends.

## 2. Rheological Behavior of Polymer/CNT Nanocomposites

### 2.1. Polycarbonate-Based Nanocomposites

Polycarbonate (PC) is a high-performance thermoplastic polymer usually exploited in a wide range of applications, such as the automotive, building and electronics fields. Since the beginning of the last decade, PC was exploited as model polymer to assess the effect of the introduction of CNTs on the rheological behavior of resulting nanocomposite systems. In fact, some research was focused on the viscoelastic response of PC/CNT nanocomposites and, especially, on the rheology/melt processing relationships, a full understanding of which allowed the optimization of the processing conditions in order to control the dispersion of CNTs in polymeric matrices [45,46,47].

Pötschke et al. [48], for the first time, reported a comprehensive study about the rheological behavior of PC/MWCNT systems, obtained by diluting a masterbatch containing 15 wt.% of nanofillers in a twin-screw extruder. The results from oscillatory linear viscoelastic measurements documented a significant increase in the complex viscosity upon increasing the nanofiller loading, mainly in the low frequency region, while the differences between the unfilled PC and nanocomposites were diminished at high frequencies due to the more pronounced shear thinning behavior of the filled materials. Moreover, nanocomposites containing less than 2 wt.% of MWCNTs exhibited a Newtonian behavior, quite similar to that of the unfilled matrix; above this critical nanofiller concentration, the complex viscosity curves showed a progressively steeper slope at low frequencies, indicating a gradually more pronounced non-Newtonian behavior as the nanofiller loading was increased. Furthermore, a concurrent increase in storage and loss moduli was recorded, with a remarkable modification of the frequency-dependence of both moduli in the low frequency range, causing the disappearance of the homopolymer-like terminal behavior exhibited by unfilled PC. All the observed peculiarities in the rheological behavior of nanocomposites containing more than 2 wt.% of MWCNTs were ascribed to a dramatic modification of the material’s microstructure upon nanofiller introduction, involving the formation of an interconnected structure of anisotropic nanofillers, which resulted in the appearance of an apparent yield stress observable in the change in the frequency-dependence of the rheological functions.

The linear viscoelastic response of PC/MWCNT nanocomposites was further investigated for a series of systems containing 23 different loadings of nanofillers, produced by melt-mixing using the masterbatch dilution method, with the aim of elucidating the possible interactions between the formed nanofiller superstructure and the physical network composed of entangled polymer chains [49]. In particular, dynamic melt rheological measurements were carried out across a wide range of temperatures, revealing a transition from a liquid-like to solid-like rheological response in correspondence with a critical nanotube loading, termed the “rheological percolation concentration”, which was associated with the formation of a nanotube network spanning the whole matrix. Viscoelastic tests performed at different temperatures documented a remarkable sensitivity of the percolation threshold toward temperature, suggesting that the observed liquid-to-solid transition did not originate exclusively from the nanotube network formation but from a combined nanofiller/polymer network affecting the mobility, characteristic structure and relaxation dynamics of the polymer chains.

**Figure 1:**
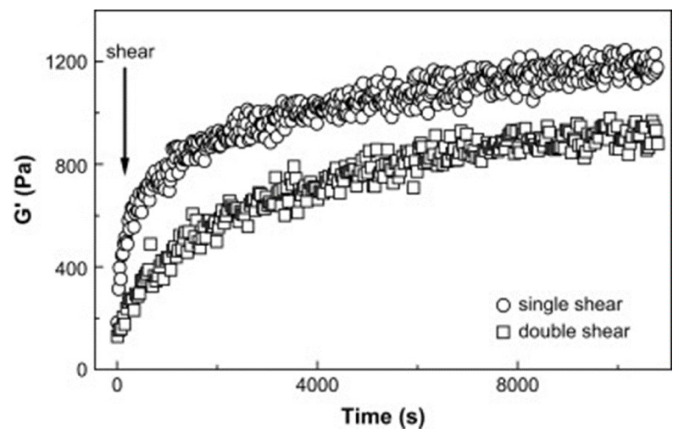
Recovery of the storage modulus versus time. Reprinted with permission from [50]. Copyright (2008) Elsevier.

The kinetics of the formation and destruction of this hybrid polymer/CNT network as a result of defined shear deformations applied to the material was deeply investigated through time-resolved measurements of the dynamic shear modulus of a PC/MWCNT nanocomposite containing 0.6 vol.% of nanofillers [50]. First, the sample was subjected to an annealing step above its glass transition temperature to achieve a defined morphology of the network. Then, transient shear was applied to the melt, following two procedures, namely a single and a double shear, involving the application of a single or a double shear pulse for 10 s, respectively. Interestingly, a recovery of the storage modulus after the application of the shear was obtained for both regimes of deformation (Figure 1); this behavior was ascribed to a mechanism of the reformation of the network of interconnected nanotube agglomerates, which was described by a combination of percolation and cluster aggregation theory. In particular, such a combined approach was primarily proposed to explain the recovery of the material’s electrical conductivity after transient shear deformation for PC/CNT composites [51,52]. Briefly, the time-dependent transition from an insulating to conductive state observed during iso-thermal annealing in the melt state was modeled using the classical theory of electrical percolation, modified through the introduction of a time-dependent concentration, accounting for the presence of CNT agglomerates, which are not considered in the percolation theory (that assumes a homogeneous dispersion of fillers within the matrix). Using this model, the growth of the conductive pathway spanning the matrix was described in terms of a clustering phenomenon, involving the formation of a network of interconnected nanotube agglomerates. Similarly, the recovery of the shear modulus after a deformation step was explained using the same approach, considering the nanotube agglomerates as “solid-like” fillers embedded in the polymer matrix.

Abbassi and co-workers [53,54] evaluated the linear rheological behavior of PC/MWCNT nanocomposites, focusing on the effect of the temperature and of the possible nanotube orientation on the polymer/filler and filler/filler interactions established within the systems. Oscillatory shear measurements showed a progressive restraint of the chain relaxation as the content of MWCNTs increased, attributed to a gradual domination of the intertube interactions leading to the formation of a percolation network that hindered the long-range motion of the polymer macromolecules. Rheological measurements performed at different temperatures allowed the discovery of a remarkable temperature-dependence of both the percolation threshold and strength of the formed network, due to the increase in the intertube interactions with temperature resulting from the enhanced cohesive energy of the network. Furthermore, the application of a shear flow prior to the measurements was found to be effective in promoting a preferential alignment of CNTs in the flow direction and caused the obtainment of lower viscosity values; this finding was explained considering that the orientation of nanofillers significantly reduced the interconnectivity between the nanotubes, giving rise to a remarkable increase in the percolation threshold.

The characteristics of the percolation network formed by CNTs are influenced also by the macromolecular architecture of the used polymer matrix. In this context, Zeiler at al. [55] formulated PC/MWCNT composites based on two different batches of PC with different average molar masses, by diluting a masterbatch through melt-mixing processing. Shear oscillatory measurements performed at different temperatures on nanocomposites containing nanofiller loadings ranging from 0.5 to 5 wt.% showed a beneficial effect of low molar masses and high temperatures on the kinetics of the formation of the CNT network. In fact, at low temperatures or in the presence of high viscosity matrices, larger stresses are generated within the polymer, thus promoting a more pronounced breakup of the formed nanofiller network.

**Figure 2:**
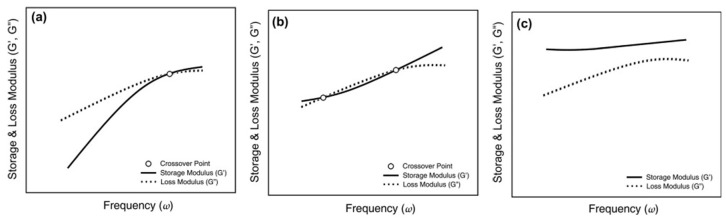
Typical storage and loss moduli of polymer/carbon nanotube (CNT) composites with different amounts of CNTs, as a function of frequency: (**a**) pure polymer, (**b**) with low CNT content and (**c**) with high CNT content. Reprinted with permission from [56]. Copyright (2014) Elsevier.

An alternative approach to calculate the rheological percolation threshold based on the crossover point (defined as the frequency at which G’ and G” moduli intersect), was proposed by Mun and co-workers [56] for a series of PC/MWCNT systems based on four PC matrices characterized by different number-average molecular weights. Small amplitude oscillatory shear tests revealed different kinds of behavior for the investigated systems regarding the crossover point between the two dynamic moduli. As reported schematically in Figure 2a, unfilled PC exhibited a single crossover point, while the introduction of low loadings of MWCNTs induced the appearance of a second crossover point at low frequencies (Figure 2b), due to the different influence that the nanofiller exerted on the trends of the storage and loss moduli. Additionally, for higher nanofiller content, the two crossover points approached each other and eventually disappeared (Figure 2c), due to the transition from liquid to pseudo-solid behavior involving a predominance of the elastic component over the whole tested frequency range. The crossover point appearing at low frequencies, being indicative of the formation of the percolative CNT network, was exploited for determining the percolation threshold of the investigated materials, defined as the lowest nanotube loading required to induce the presence of a low-frequency crossover at the reference frequency of 1 rad/s.

### 2.2. Polyethylene-Based Nanocomposites

Currently, polyethylene (PE) is largely exploited in a huge range of industrial applications, including the automotive and packaging fields, mainly due to its versatile processability and low cost. However, due to the increasing demand for innovative technological materials, the interest in PE-based nanocomposites containing CNTs is steadily increasing, particularly concerning their rheological behavior, the evaluation of which has usually been exploited to inspect the dispersion extent of the embedded nanofillers within the PE.

Zhang et al. [57] studied the linear rheological behavior of a series of high-density polyethylene (HDPE)-based nanocomposites containing different amounts of single-walled carbon nanotubes (SWCNTs) ranging from 0.5 to 5 wt.%. A two-step processing method was employed for the formulation of the nanocomposites, involving a preliminary adsorption of SWCNTs onto the surface of PE powder (achieved by spraying an aqueous solution containing the nanofillers directly onto the polymer), followed by a melt-mixing step in a twin-screw extruder. Results from dynamic frequency sweep measurements showed a sudden change in the material rheological behavior as the SWCNT loading exceeded 1.5 wt.%, indicating the onset of solid-like behavior and, hence, a rheological percolation threshold at that nanofiller content. More specifically, unlike the unfilled matrix that showed typical homopolymer-like terminal behavior, for nanocomposites containing SWCNT loading higher than 1.5 wt.%, the dependence of both the storage and loss moduli on frequency became weak, suggesting that large-scale polymer relaxation was effectively restrained by the presence of the nanoscale network structure formed by the embedded fillers. Once this network was formed, a further increase in the nanofiller content did not promote significant modifications of the rheological properties of the nanocomposites.

**Figure 3:**
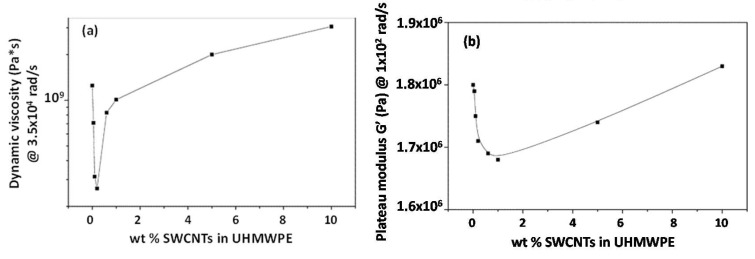
Variation of (**a**) dynamic viscosity and (**b**) plateau modulus as a function of nanotube content in ultra-high-molecular-weight polyethylene (UHMWPE)/single-walled carbon nanotubes (SWCNT) nanocomposites. Reprinted with permission from [58]. Copyright (2006) American Chemical Society.

A similar processing strategy, encompassing the preparation of a nanofiller-coated polymer powder and a subsequent solvent casting step, was exploited by the same research group to formulate ultra-high-molecular-weight polyethylene (UHMWPE)/SWCNT nanocomposites, which were characterized through small amplitude oscillatory shear tests [58]. A rather unusual rheological behavior was observed, involving the appearance of a minimum in the trend of the complex viscosity and storage modulus, located in the low frequency region, as a function of the SWCNT content. In particular, as depicted in Figure 3, the complex viscosity exhibited a distinct minimum at 0.1–0.2 wt.% of SWCNTs, while the plateau modulus decreased up to about 1 wt.%, after which it increased. The presence of the minimum in the viscosity trend was explained considering a selective adsorption of the high molar mass fraction of UHMWPE onto the nanotube surfaces. In fact, the selected matrix has a broad molar mass distribution, and the macromolecules constituting the high molar mass fraction, because of the van der Waals interactions, tend to be adsorbed on the nanotubes. In this way, the polymer forming the matrix bulk (i.e., not immobilized onto the nanotube surface) will have a low average molecular weight, thus causing a decrease in the entanglement density and faster relaxation processes, resulting in lower viscosity values. To confirm the reduced apparent molecular weight of the matrix in the bulk, the trend of the loss angle as a function of frequency was evaluated for nanocomposites: lower modulus values with respect to the unfilled UHMWPE were observed. Upon increasing the amount of SWNTs from 0 to 0.1 wt.%, the phase angle increased, indicating less pronounced elastic behavior of the nanocomposites with respect to that of the unfilled polymer. For the nanocomposites containing high SWCNT loading, both the viscosity and storage modulus increased, because of the formation of interconnected structures of the embedded anisotropic nanofillers, causing the appearance of an apparent yield stress behavior.

An analogous behavior was documented by Vega et al. [59] for an HDPE-based nanocomposite obtained by the melt-mixing the selected matrix with an in situ-polymerized HDPE/SWCNT masterbatch. The results collected through frequency sweep measurements suggested the absence of a percolative network in the nanocomposite, as in the investigated frequency range, both the storage modulus and complex viscosity significantly decreased as compared to those for the unfilled matrix. Furthermore, the absence of a percolation event was confirmed by the Cox–Merz rule (predicting the equivalence of the steady shear viscosity and the complex viscosity, when the steady shear rate coincides with the frequency), which usually holds for entangled melts and composites below the percolation threshold. The observed reduction of the rheological functions was attributed to the adsorption of polymer chains with longer relaxation times onto the nanotube surface and to the consequent decrease in the entanglement concentration in the remaining polymer. In other words, once the macromolecules are adsorbed onto SWCNTs’ surface, they become inactive within the matrix, no longer contributing to the viscosity or to the average relaxation time of the system. As a result, the system was characterized by an increased mobility that affected the extensional behavior too; more specifically, the nanocomposite exhibited a lower elongational viscosity and melt strength as compared to the neat matrix, probably because of the decreased number of entanglements.

The phenomenon of the selective adsorption of PE macromolecules onto the nanotube surface was thoroughly investigated by Patil et al. [60] for a linear PE with a broad molar mass distribution, filled with different loadings of SWCNTs. Linear rheological measurements conducted at different temperatures revealed a temperature-dependent decrease in viscosity for the nanocomposites with respect to the unfilled matrix, in the high-frequency region. More specifically, the viscosity drop, attributed to the selective adsorption of high-molar-mass chains to the embedded nanofillers, was observed when the measurements were carried out at lower temperatures but disappeared at high temperatures, confirming a key role of the relaxation time of the polymer macromolecules on the adsorption mechanism.

Xiao et al. [61] studied the rheological behavior of a series of nanocomposites based on low-density polyethylene (LDPE) and containing up to 10 wt.% of multi-walled carbon nanotubes (MWCNTs), through both steady shear and small amplitude oscillatory shear measurements, aiming at verifying the validity of the Cox–Merz rule for the formulated systems. For LDPE-based composites, the superposition of the two viscosity curves was observed at low MWCNT loadings but became inaccurate for the nanocomposites containing high nanofiller contents, for which a distinct shear-thinning behavior appeared in the viscosity curves. This feature implies that the Cox–Merz rule cannot be applied when the rheological behavior of the nanocomposites changes from liquid-like to predominantly solid-like. To gain a better understanding of the liquid-to-solid transition experienced by nanocomposites at high nanofiller loadings, the trend of tanδ (where δ is the phase angle) as a function of frequency was evaluated, this rheological property being very sensitive to the microstructural modification occurring in complex polymer-based systems. tanδ showed a decreasing trend with increasing the nanofiller content, revealing a progressively more pronounced elastic-like response for the nanocomposites and a gradual fluid–solid transition, which was associated with a gelation phenomenon occurring in the system. This gelation event was characterized according to the Winter criterion, predicting the independence of tanδ from frequency at the gel point; besides, it was found to occur for a nanofiller content of 4.8 wt.%, at which, as supposed by the authors, the embedded MWCNTs are likely to organize in a continuous network spanning the whole matrix.

Apart from the intrinsic characteristics of CNTs and of the interactions established between the matrix and the nanofiller, the macromolecular architecture of the polymer, in terms of the molar mass and content of long and/or short branches, also plays an important role in determining the rheological behavior of the nanocomposite materials. In this context, Vega et al. [62] studied the influence of chain branching and molecular weight on the melt rheology of PE/MWCNT nanocomposites obtained by melt-mixing, using three PE samples differing in terms of molecular weight and branching. A preliminary rheological characterization performed on the unfilled matrices revealed higher viscosity and storage modulus values for the sample with a high molar mass; furthermore, due to the polydispersity of the selected PEs, the terminal region was not entirely reached. Conversely, the creep time and the recoverable compliance were not remarkably affected by the different macromolecular architecture of the samples. As far as the nanocomposites are concerned, the viscoelastic response was strongly dependent on the polymer matrix; more specifically, for systems based on linear low-molecular-weight PE and on a long branch-containing polymer, the viscous flow region was delayed at low frequencies, resulting in the appearance of a plateau and of a shoulder in the storage modulus curves, indicating a distinct enhancement of the elastic character of these systems. Conversely, the rheological behavior of the nanocomposite based on the high-molecular-weight PE seemed quite unaffected by the addition of nanofillers. The obtained results indicated that the molecular weight of the matrix, rather than the formed nanotube network, is the main factor controlling the rheological response of the systems. More specifically, in the case of a matrix with a high molecular weight and broad molar mass distribution, the effects caused by the nanotube network were screened, and the viscoelastic behavior of the nanocomposite was determined by the superposition of the polymer network with the nanofiller percolative superstructure.

A further investigation regarding the possible influence of the macromolecular characteristics of the polymer on the nanocomposite’s rheological functions was performed by Nobile et al. [63], on MWCNT-containing nanocomposites based on two kinds of HDPE, differing in terms of average molar mass and polydispersity index. Large amplitude oscillatory shear tests demonstrated a decrease in the upper limit of the linear viscoelastic region for the nanocomposites as compared to the unfilled matrices, irrespectively of the macromolecular architecture of the polymers. Similarly, the results of small amplitude oscillatory shear measurements, arranged in a van Gurp–Palmen plot (representing the trend of the phase angle as a function of the complex modulus), indicated the formation of a percolation network for a content of MWCNTs of about 2 wt.%, although no remarkable differences were noticed for the nanocomposites based on the two PE samples. This finding was associated with the fact that the molar masses of the two selected matrices are not different enough to induce significant modifications in the nanofiller/matrix interactions, thus resulting in similar levels of PE/MWCNT interconnection.

The achievement of the rheological percolation threshold in a CNT-filled polymer is strongly dependent on the intrinsic properties of CNTs, including their surface properties, the possible presence of waviness or coils and, above all, their aspect ratio; in fact, the formation of the percolation network is strictly related to the interconnectivity ability of the nanotubes, which, in turn, is determined by their aspect ratios [64]. With the aim of verifying if the changes in the CNT geometry are capable of promoting significant modifications of the rheological behavior of UHMWPE-based nanocomposites, MWCNTs were subjected to a sonication treatment for different time intervals and then introduced in the matrix through a two-step processing method, encompassing mechanical mixing and hot compaction [65]. Preliminary characterizations performed on the treated nanotubes revealed a progressive decrease in the MWCNTs’ average hydrodynamic diameter with increasing sonication time. The results of linear viscoelastic measurements showed a detrimental effect of the sonication-induced shortening of nanotubes on the formation of the percolation network, since the lower aspect ratios of the nanofillers treated for long sonication times prevented the formation of a percolation pathway at the selected nanotube loading. As a consequence, due to the decreased ability of shorter nanotubes to restrain the polymer chain dynamics, the rheological response of the nanocomposites containing MWCNTs sonicated for large time intervals was very similar to that of the unfilled matrix. This finding was further confirmed through stress relaxation measurements; in particular, untreated nanotubes caused an incomplete relaxation of the polymer chains, giving rise to the appearance of a pseudo-solid-like behavior. Differently, the nanocomposite containing MWCNTs sonicated for long times showed a liquid-like relaxation behavior quite similar to that of the unfilled polymer, indicating that the embedded treated nanofillers were not able to affect the relaxation spectrum of UHMWPE.

**Figure 4:**
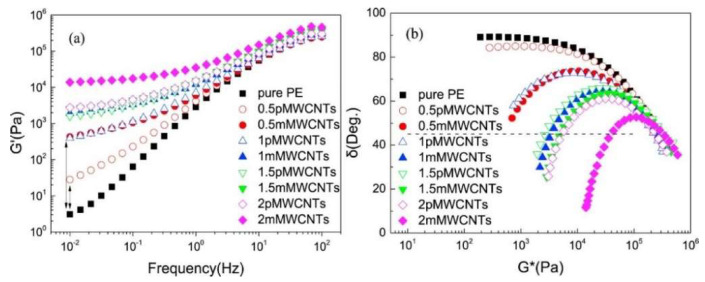
(**a**) Dynamic storage modulus (G′) and (**b**) v-GP plots for the composites with various unmodified (pMWCNTs) and functionalized (mMWCNTs) nanofiller loadings. Reprinted with permission from [66]. Copyright (2019) Elsevier.

A commonly exploited strategy to improve the CNT/matrix interactions at the interface involves the chemical functionalization of nanotubes with different chemical groups promoting a better level of dispersion of the nanofiller and an enhanced affinity towards the polymer matrix. In this context, Peng and co-workers [66] modified MWCNTs with a cationic waterborne epoxy emulsion; briefly, pristine MWCNTs were first incorporated in a mixture containing the cationic aqueous epoxy emulsion, stirred at room temperature, filtered and washed several times with distilled water, then finally vacuum dried. The as-functionalized nanofillers were melt-mixed with HDPE, and the effectiveness of the functionalization strategy in enhancing the interfacial interactions between nanofillers and polymer chains was verified by evaluating the linear rheological behavior of the nanocomposites. Rheological analyses revealed that a more effective nanotube network was developed at a lower content of functionalized CNTs as compared with that of unmodified nanofillers. More specifically, as shown in Figure 4a, the storage modulus of the materials containing functionalized nanotubes was higher than that of the corresponding nanocomposites containing the same amount of unmodified nanofillers, and the plateau modulus, resulting from the liquid-to-solid transition, was more evident, suggesting a more effective restraint of the large-scale polymer relaxation. The percolation threshold of the two series of nanocomposites was detected using the van Gurp–Palmen plot, depicted in Figure 4b; for functionalized CNT-based systems, a clear variation of the rheological behavior was observed for filler contents above 0.5 wt.%, while higher loadings of unmodified nanotubes were needed to initiate a similar modification.

An innovative time–concentration superposition principle was proposed by Song and co-workers [67] to investigate the hydrodynamic-to-non-hydrodynamic transition experienced by HDPE/MWCNT nanocomposites. In particular, a normalization of both storage and loss moduli using a frequency-dependent critical nanofiller content was performed, with the aim of properly evaluating the role of chain dynamics in the bulk phase on the liquid-to-solid transition usually observed in CNT-containing systems. More specifically, the application of the time–concentration superposition principle allowed the relation of the rheology of the materials at high frequency to that at low filler loadings and vice versa, revealing that the elasticity of the nanocomposite materials in the non-hydrodynamic regime follows the prediction for the reaction-limited cluster–cluster aggregation model, involving the formation of a strongly linked network dominated by individual flocs of nanofillers.

Recently, a new approach based on the evaluation of the dynamic rheological data for a HDPE/MWCNT nanocomposite was proposed to determine the super-toughness point and to predict the electrical percolation threshold of the materials [68]. In particular, a correlation between the mechanical properties and rheological behavior was established, allowing the correlation of the rheological gel-point, corresponding to the formation of a nanofiller superstructure, to the toughness of the nanocomposite; in this way, the occurrence of super-toughness at the gel point, originating from the elastic deformation and eventual destruction of the CNT network, was demonstrated.

### 2.3. Polypropylene-Based Nanocomposites

Polypropylene (PP) is a commodity thermoplastic, largely employed in the packaging and automotive fields; its functional performance, such as electrical and/or thermal properties, can be remarkably improved through the incorporation of CNTs. Therefore, extensive research was conducted on PP/CNT materials, mainly focused on the exploitation of different processing strategies and on the application of external stress fields to improve the dispersion of CNTs within the polymer [69,70]. In this context, the evaluation of the rheological properties of the produced materials allowed the verification of the effectiveness of the proposed methods in obtaining tailored engineered polymer/nanofiller interfaces, hence promoting the achievement of superior properties.

As an example, Menzer and co-workers [71] evaluated the effect of a ball-milling treatment on the dispersion and percolation behavior of MWCNTs in melt-mixed nanocomposites based on isotactic polypropylene. Preliminary analyses of the structural characteristics of nanotubes found a remarkable reduction in the nanofiller length after the ball-milling and a more compact primary agglomerate morphology compared to that of untreated nanotubes. These structural modifications significantly affected the rheological behavior of the obtained nanocomposites; in fact, MWCNTs subjected to ball-milling exhibited higher values of the rheological percolation threshold, along with lower storage moduli and viscosities, with respect to untreated nanotubes. The observed differences in the rheological behavior were ascribed to the highly compact agglomerates formed by the MWCNTs subjected to ball-milling, which limited the formation of an extended nanofiller/polymer contact area, and to the reduced length of the treated nanofillers, causing a decrease in the entanglements formed with the polymer chains.

**Figure 5:**
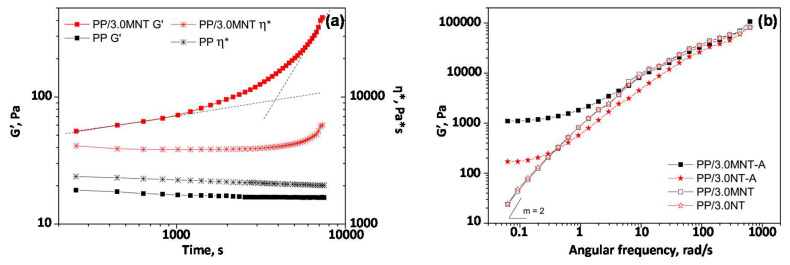
Time evolution of storage modulus and complex viscosity (**a**), and storage modulus as a function of frequency (**b**) for polypropylene (PP)-based nanocomposites (NT: pristine MWCNTs; MNT: surface modified MWCNTs; PP/3.0NT-A and PP/3.0MNT-A: PP-based composites containing pristine and modified MWCNTs, respectively, subjected to annealing). Adapted with permission from [72]. Copyright (2014) Elsevier.

Gentile et al. [72] combined a thermal treatment with a surface modification of MWCNTs, involving a mechanochemical functionalization with maleic anhydride PP, to enhance their extent of dispersion within a PP matrix; the linear rheological behavior of the resulting composites was investigated to verify the effectiveness of the proposed strategy. In particular, the temporal evolution of the storage modulus and complex viscosity at 200 °C was recorded for the unfilled matrix and the nanocomposite containing 3 wt.% of modified nanotubes (Figure 5a).

A steadily decrease in the rheological functions, ascribed to the occurrence of some degradation phenomena during the thermal treatment, was observed for neat PP. Conversely, the nanocomposite exhibited a gradual increase in both viscoelastic properties, and, especially for the storage modulus trend, a steep slope was recorded at high treatment times. To deeply investigate this behavior, frequency sweep tests were performed on nanocomposite samples containing both unmodified and functionalized MWCNTs, before and after the thermal treatment. The storage modulus curves, shown in Figure 5b, confirmed the beneficial effect of the annealing step in favoring the formation of a percolation network, since the terminal behavior exhibited by both systems before the thermal treatment disappeared after the annealing step: this finding indicated the occurrence of a percolative event promoted by the thermal treatment. More specifically, the observed behavior was ascribed to the occurrence of a thermally activated flocculation process, involving the dynamic reassembly of nanofiller clusters into a space-spanning network due to the inter-nanotube attraction.

Linear and nonlinear rheological characterization, both in shear and elongational flow, was performed by Fernandez et al. [73] on PP/MWCNT nanocomposites. The latter were irradiated with an electron beam following two different procedures, namely (i) the irradiation of the nanocomposite after the extrusion step or (ii) the irradiation of the unfilled matrix only, before the processing. A remarkable influence of the modification of the PP macromolecular architecture resulting from irradiation on the rheological behavior of nanocomposites was documented, especially when the systems were subjected to the elongational flow. In particular, the unfilled matrix and irradiated nanocomposites showed a well-pronounced strain-hardening behavior, due to the presence of long chain branches, whose formation was induced by irradiation. Surprisingly, in the nanocomposites based on irradiated matrix, strain hardening was not observed, because of the occurrence of a conformational change in the branched structure during the extrusion, involving an alignment of the long branches to the backbone chains. Additionally, the thermal-reversibility of this behavior was confirmed, since a thermal treatment performed at high temperatures after the extrusion allowed the recovery of the original branch configuration, as verified by the appearance of a strain-hardening phenomenon during a further rheological characterization performed after annealing.

Apart from the presence of branched structures, the rheological properties of PP/CNT nanocomposites strongly depend on the molecular weight of the selected polymer matrix. Teng and co-workers [74] exploited three different PP matrices, characterized by different average molar masses and melt flow indices, to produce melt-mixed nanocomposites containing up to about 9 wt.% of MWCNTs. To simulate the behavior of the materials during processing, linear rheological measurements were performed in a capillary rheometer. The obtained flow curves showed different behaviors depending on the value of shear rate; particularly, at the lowest tested shear rate values, the viscosity of the nanocomposites increased with increasing CNT loading, irrespectively of the PP molar mass, due to the confinement of the polymer chains in the presence of the embedded nanofiller. On the other hand, at higher shear rates, the differences between nanocomposites and the corresponding matrices were less evident, since the shear thinning behavior and the wall slip phenomenon were predominant and the material viscosity was almost unaffected by the nanotube content. For the nanocomposites based on PP with the highest molar mass and the lowest melt flow index, the drop in viscosity in the high shear rate region was more pronounced, and the nanocomposites containing low amounts of MWCNTs showed lower viscosity values, as compared to the unfilled matrix. This finding was attributed to the higher viscosity of the PP matrix, which involved higher shear stresses, able to induce a preferential orientation of the nanotubes during the flow into the capillary, causing a decrease in the nanocomposite’s flow resistance.

In some cases, the study of the viscoelastic characteristics of PP/CNT systems was used to formulate different models for the simulation of the material behavior during processing. In this context, Thiébaud et al. [75] performed an accurate evaluation of the linear rheological behavior of PP/MWCNT systems to build a consistent model, suitable for simulating the flow of the material during the processing in a twin-screw mixer. Rheological tests were carried out in a capillary rheometer across a wide shear rate range (10^−1^–10^4^ s^−1^) and at different temperatures, revealing a progressive disappearance of the Newtonian plateau in the flow curves and a concurrent occurrence of a more pronounced shear-thinning behavior as the content of MWCNTs was increased. The differences in the viscosity curves were more significant at low shear rates, while at higher shear rate values, the shear-thinning and the wall-slip phenomena occurring between the materials and the wall of the rheometer became predominant, causing a minimization of the effects due to the nanofiller content. The obtained rheological data were employed for building a constitutive model based on an extension of the Carreau law, coupled with a thermal factor accounting for the variation of the temperature-induced viscosity change, which was employed to perform finite element analysis of the system flow; in this way, it was possible to compute the velocity and the shear rate during the mixing.

**Figure 6:**
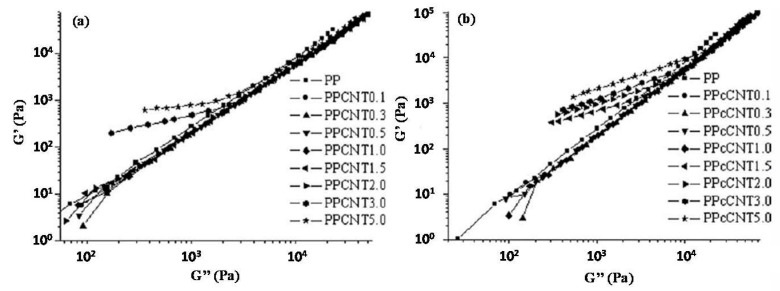
Han plot for (**a**) pristine and (**b**) purified CNT-containing nanocomposites (CNT: pristine nanotubes, cCNT: purified nanotubes). Reprinted with permission from [77]. Copyright (2012) John Wiley and Sons.

When the surface of the CNTs is modified through functionalization procedures, a consequent change in the characteristics of the matrix/nanofiller interfacial region occurs, strongly affecting the dynamics of the polymer macromolecules and the relaxation behavior of the nanocomposite materials [76]. The stress relaxation behavior of PP-based nanocomposites containing unmodified and purified MWCNTs was assessed by Zhou and co-workers [77], who documented an increase in the linear relaxation modulus with increasing nanotube loading and the appearance of a plateau at long time scales for the systems containing nanofiller content above the percolation threshold, attributable to the incomplete relaxation of the PP macromolecules due to the formed polymer/nanotube hybrid network. The formation and the characteristics of this network superstructure were studied using the Han plot shown in Figure 6.

This kind of representation of the rheological data is usually utilized to investigate the temperature-induced modifications of the internal microstructure of polymer blends or block copolymers, but it can be also used to elucidate microstructure evolution in polymer nanocomposites at a fixed temperature. First, the reaching of the percolation threshold caused a divergent behavior in the terminal region; in fact, the curves of the nanocomposites below the percolation threshold superimposed with the curve of unfilled matrix, while the formation of the nanofiller network induced a decrease in the curve slope, due to the enhancement of the material heterogeneity. It is noteworthy that the introduction of the modified MWCNTs caused an anticipation of the percolation event, as compared to the unmodified nanofillers. Furthermore, the inflection point was shifted toward higher frequency values with an increasing of the nanotube content, indicating an increased level of physical association within the nanocomposites at high nanofiller loading, which required higher energy to modify the degree of heterogeneity of the materials.

The influence of the aspect ratio of the MWCNTs on the rheological behavior of PP-based nanocomposites was evaluated by Pan et al. [78], by introducing a concept of “gel-like behavior” to investigate the nanofiller network formation. Dynamic rheological measurements revealed the typical transition from liquid-like to solid-like rheological behavior with increasing the MWCNT content and allowed the calculation of the percolation threshold, which was dependent on the nanofiller aspect ratio, with higher aspect ratio nanotubes leading to a lower percolation value. Furthermore, the Winter–Chambon method was used to determine the gel content and strength of the formed network, through the application of a power-law describing the shear relaxation modulus of critical gels:

(1) $$G\left(t\right)=S_{g}t^{-n}$$

where *S_g_* is the gel strength and *n* is related to the fractal dimension of the network. More specifically, the exponent n reflects the degree of the compactness of the network: a small value indicates the formation of a more elastic gel, while high values are typical of physical gels, exhibiting weak strength. Lower *n* values and higher *S_g_* were obtained for high-aspect-ratio MWCNTs, demonstrating the better effectiveness of these nanofillers in increasing the strength of the MWCNT network in the polymer matrix.

An unusual thermo-rheological behavior was observed for PP nanocomposites containing higher contents (up to 30 wt.%) of MWCNTs, with an increase in both complex viscosity and storage modulus as the temperature increased from 190 to 220 °C [79]. For these systems, a negative value of the flow activation energy, accounting for the energy barrier to polymer segmental motion, was found, implying an inverse dependence of the viscoelastic properties on temperature. The thermo-rheological behavior of the nanocomposites was evaluated using the van Gurp–Palmen plot, revealing a thermo-rheological simplicity for unfilled PP and nanocomposites containing low MWCNT amounts; conversely, the nanocomposite containing 30 wt.% of nanotubes showed a complex thermo-rheological behavior, arising from a difference in the temperature’s dependence on polymer motion between the bulk polymer chains and the macromolecules located at the interphase.

### 2.4. Polystyrene-Based Nanocomposites

Polystyrene is a versatile thermoplastic polymer commonly employed in a variety of commercial applications, such as food packaging, electronics, the automotive industry and leisure. The general behavior reported in the literature concerning the rheological response of polystyrene (PS)/CNT nanocomposites involves the obtainment of higher viscosity and modulus values as a function of nanofiller loading and a transition from liquid-like to solid-like behavior as the CNT loading increases, accounting for the formation of a semi-3D nanofiller network that strongly affects the relaxation dynamics of PS chains [80,81,82,83,84]. A different rheological response was found by Amr et al. [85] in the case of nitric acid-treated CNT-containing nanocomposites. In particular, a decrease in complex viscosity was recorded for nanocomposites containing 0.1 and 0.5 wt.% of treated CNTs, with respect to the unfilled matrix; this unusual behavior was associated with the lack of inter-tube interactions and with the plasticizing action of the embedded nanofillers at such low loadings.

A model monodisperse PS was used by Mitchell and co-workers [86] to produce nanocomposite materials containing unmodified and functionalized SWCNTs, prepared by the in situ generation and reaction of organic diazonium compounds. Melt-state linear viscoelastic measurements were exploited to investigate the state of dispersion of both nanofillers, revealing a lower percolation threshold for functionalized nanotubes because of their improved compatibility as compared to unmodified SWCNTs, explaining their better dispersion. More specifically, rheological data were collected across a range of temperatures and superposed using the time–temperature superposition principle to obtain viscoelastic mastercurves. Significant modifications were observed for functionalized nanofillers in the terminal region, involving a frequency-independent behavior of the storage modulus and the development of a finite yield stress, causing a divergence in the complex viscosity vs. complex modulus plot, due to the interference of a percolated filler structure. This behavior was also observed for unmodified SWCNTs, though at higher contents with respect to those in the functionalized ones, consistent with the poorer dispersion of nanofillers in the latter case.

An interesting study by Kota et al. [87] was focused on a quantitative characterization of an interpenetrating phase polymer nanocomposite, formed by the percolation of MWCNTs in a PS matrix, through melt rheology. To this end, the PS/MWCNT system was treated as a combination of two phases: a continuous PS phase reinforced by non-interacting MWCNTs and a continuous phase consisting of the solid-like network of percolated nanotubes. First, the degree of percolation (derived from electrical conductivity data) was used to assess the contribution of each phase to the dynamic rheological properties of the nanocomposite, allowing the determination of the properties of the continuous MWCNT path and the isolation of the effect of non-interacting nanofillers dispersed in PS. Then, the nanotube network was characterized in terms of oscillatory response at low and high frequencies, disclosing a peculiar behavior indicative of a scaffold-like microstructure that showed a stick-slip friction mechanism at the interface of the percolated nanotube network at higher frequencies.

McClory and co-workers [88] investigated the effect of screw speed on the rheological percolation of PS/MWCNT nanocomposites containing a wide range of nanofiller loadings, produced in a twin-screw intermeshing co-rotational extruder. In general, a progressive increase in the complex viscosity, storage modulus and inverse of tanδ was observed with increasing screw speed; however, as indicated by the morphological characterization performed on composite samples, using screw speeds higher than 100 rpm (together with shorter residence times) was not effective for disentangling the primary nanofiller agglomerates. Interestingly, for the nanocomposite containing 5 wt.% of MWCNTs, the percolation threshold was reached at any selected screw speed, indicating that for a high content of nanotubes, a broad distribution of MWCNT dispersion (consisting of micrometric agglomerates, sub-agglomerates and individual tubes) was obtained for all the selected screw speeds. This observed arrangement of MWCNTs within the host matrix is sufficient, from a rheological point-of-view, to alter polymer dynamics.

The effect of different processing methods on the rheological behavior of PS/MWCNTs was evaluated by Faraguna et al. [89] for a series of nanocomposites based on unmodified and modified nanotubes with phenyl propane ester and polystyrene functional groups. More specifically, two PS matrices differing in terms of average molar mass and several MWCNTs modified according to different functionalization procedures were combined to produce nanocomposite materials through melt- and solution-mixing. The results obtained through linear viscoelastic measurements revealed that solution-mixed systems exhibited improved complex viscosities and lower percolation thresholds with respect to their melt-mixed counterparts, accounting for a general better dispersion of nanofillers and higher length of MWCNTs.

**Figure 7:**
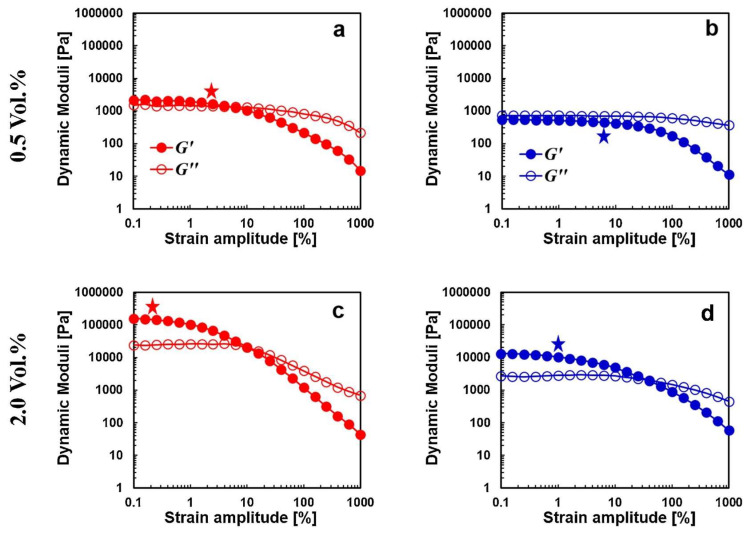
Large amplitude oscillatory shear (LAOS) response of polystyrene (PS)/MWCNTs nanocomposites: (**a**,**c**) solution-mixed and (**b**,**d**) melt-mixed nanocomposites. The star symbols show the critical strain amplitudes. Reprinted with permission from [90]. Copyright (2020) Elsevier.

A similar approach was followed by Kamkar and co-workers [90] for PS/MWCNTs produced through solution- and melt-mixing; in this case, the effectiveness of the exploited processing strategies was evaluated in terms of the material non-linear rheological response. As shown in Figure 7, in the linear viscoelastic region, solution-mixed nanocomposites exhibited an elastic-dominant response as indicated by the larger storage modulus as compared to the loss one; conversely, melt-mixed materials showed a prevailing viscous behavior. This finding, coupled with the lower critical strain amplitude for the beginning of the non-linear regime, indicated a stronger and denser interconnected network of MWCNTs in solution-mixed samples with respect to that in melt-mixed materials.

Marcourt et al. [91] compared the rheological behavior of CNT-containing nanocomposites based on either pure amorphous or rubber-modified polystyrene. For both series of composites, a progressive increase in the storage modulus was observed as a function of nanofiller loading, with the appearance of a plateau, accounting for the inhibition of the macromolecule relaxation due to the formation of a percolation network, in the low frequency region. The increase in the storage modulus at low frequencies was described with a percolation law:

(2) $$G_{p}\approx G_{0}{\left(\Phi_{0}-\Phi_{c}\right)}^{t}$$

where $G_{p}$ is the plateau (equilibrium) storage modulus and $\Phi_{c}$ is the percolation threshold. Quite similar values of the percolation threshold were found for both series of nanocomposites, although the rubber modified PS-based system exhibited higher $G_{p}$ values and lower values of the exponent *t*, indicating a different structure of the network in the two matrices. Furthermore, elongational rheological measurements were performed, revealing a possible competition between the destruction and structuring of the nanofiller network during extensional deformation. More specifically, for CNT contents up to 1 vol.%, all the investigated materials followed the Trouton law, predicting a value of elongational viscosity three times higher than that of the shear viscosity. However, rubber-modified PS-based systems showed a remarkable deviation from this behavior for nanofiller amounts above 1 vol.%, suggesting the occurrence of some segregation phenomena that promoted the formation of connections between the aggregates, strongly reducing the mobility of polymer chains.

### 2.5. Polyamide-Based Nanocomposites

Polyamides (PA) are a class of engineering thermoplastics widely used in a large number of commercial applications, mainly due to their high strength, elasticity, toughness and abrasion resistance. Since PA/CNT composites are usually produced through melt processing, the characterization of the rheological behavior of these systems is performed to gather important information about the processing conditions, thus allowing the achievement of high-performing composites.

Wang et al. [92] carried out dynamic oscillatory measurements on PA1010-based composites containing different loadings of MWCNTs, unveiling the usual rheological behavior of thermoplastic polymers filled with anisotropic nanofillers: a remarkable increase in complex viscosity and dynamic moduli, with the disappearance of the terminal behavior marking the transition from liquid-like to solid-like viscoelastic response, was observed once the percolation threshold was reached. To gain more insight into the processability of the materials, the steady shear response of the MWCNT-containing composites was recorded, and the obtained data were fitted with the well-known Carreau model:

(3) $$\eta =\frac{\eta_{0}}{{\left[1+{\left(\dot{\gamma }t_{1}\right)}^{2}\right]}^{\left(1-n\right)/2}}$$

where $\eta_{0}$ is the zero-shear viscosity, $\dot{\gamma }$ is the shear rate, $t_{1}$ is the characteristic time and *n* is a dimensionless parameter accounting for the slope of the flow curve in the power-law region. The introduction of MWCNTs caused a dramatic increase in the zero-shear viscosity and a decrease in the exponent *n*, implying a more pronounced shear-thinning behavior at high shear rates. Furthermore, an anticipated transition from a Newtonian plateau to a shear-thinning region was observed for nanocomposites as compared to unfilled matrix, suggesting that the applied shear stress was able to align the nanotubes along the flow direction.

A different behavior was observed for PA6/MWCNTs, in which the introduction of low contents (from 0.2 to 0.5 wt.%) of nanotubes induced a decrease in the complex viscosity with respect to the unfilled matrix [93]. This finding was explained by the formation of a viscous surface layer surrounding the embedded nanofillers, thus causing an increase in the free volume that facilitated the flow of the polymer macromolecules. A further increase in the MWCNT loading resulted in higher viscosity values, due to the improved physical interactions between neighboring nanotubes or between polymer chains and nanotubes, which governed the rheological response of the materials. Furthermore, the effect of temperature on the nanocomposite’s viscoelastic behavior was probed, unveiling that the incorporation of nanofillers resulted in a higher activation energy for the polymer flow process.

The quite recent introduction of additive manufacturing technologies has stimulated growing attention toward research for polymer-based materials suitable for this specific processing; in this context, PA12 is the most common polymer used for laser sintering, mainly due to its ease of processing [94,95,96]. Bai et al. [97] characterized the rheological behavior of a PA12 powder coated with low contents of CNTs to verify the possible influence of the nanofillers on the polymer processability. Compared to the unfilled matrix, PA12/CNT systems exhibited higher complex viscosities and dynamic moduli, although the loading of the introduced nanofillers was not high enough to induce significant modifications of the trend of the matrix storage modulus and complex viscosity. Surprisingly, a remarkable increase in the viscosity as a function of temperature was recorded for all the investigated materials; this phenomenon was attributed to the incomplete melting of some PA12 particles inside the material, which melted during the measurement, causing an increase in the entanglement content and a consequent rise in the viscosity values.

### 2.6. Biopolymer-Based Nanocomposites

In this section, the main achievement concerning the rheological characterization of CNT-containing nanocomposites based on biopolymeric matrices will be discussed, including some results obtained for either biodegradable or renewable source-derived polymers, such as polylactide and poly(caprolactone).

Park et al. [98] investigated the effect of very low loadings (from 0.02 to 0.2 wt.%) of MWCNTs on the microstructure of nanocomposites systems based on a polylactide (PLA) matrix, through linear dynamic measurements. The nanocomposite materials showed higher complex viscosities as compared to unfilled PLA, and well pronounced non-Newtonian behavior across the whole investigated frequency range. Besides, a deviation from the typical behavior of an isotropic melt was observed in the plot of storage modulus vs. loss modulus, implying an increase in the heterogeneity of the material as a result of CNT incorporation and a conformational change of the PLA chains due to a physical association of nanofillers within the matrix, occurring during processing.

A very similar behavior was demonstrated by Xu et al. [99] for a series of PLA-based nanocomposites containing functionalized MWCNTs obtained through the covalent bonding of five-armed star polylactide molecules onto nanofillers by a “grafting to” method. In this case, the introduction of functionalized nanotubes caused a dramatic modification of the low frequency relaxation of PLA chains, as a consequence of the interference of the MWCNT percolated network with the long-range motion of polymer macromolecules.

**Figure 8:**
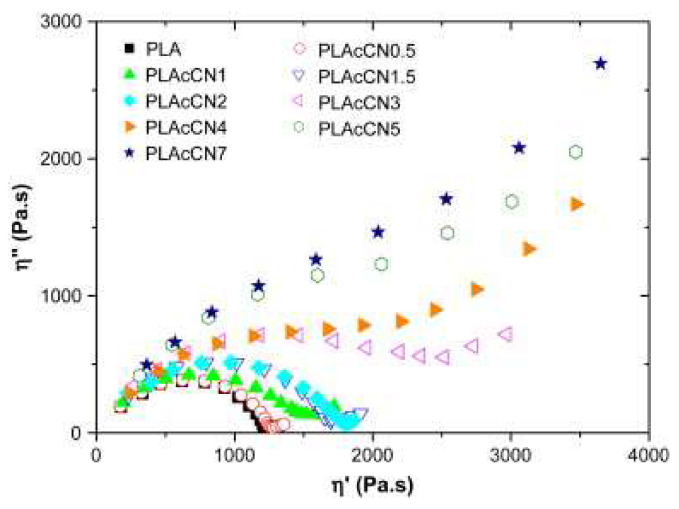
Cole–Cole plot for polylactide (PLA) and carboxylic functionalized MWCNT/PLA nanocomposites. Reprinted with permission from [100]. Copyright (2008) Elsevier.

The effect of the chemical grafting of carboxyl or hydroxyl functionalities on the nanotube surface on the linear viscoelastic behavior of PLA/MWCNT nanocomposites was studied by Wu and co-workers [100], through oscillatory measurements. The obtained results showed that both kinds of functionalized nanotube-containing materials exhibited the typical solid-like viscoelastic response, although carboxyl-modified nanofillers revealed a lower percolation threshold, suggesting a better affinity with the matrix, as compared to the hydroxyl-modified MWCNTs. Furthermore, the calculated relaxation time of the nanocomposites progressively increased with nanofiller loading, indicating that the presence of embedded MWCNTs, irrespectively of their specific functionalization, remarkably restricted the chain mobility of PLA macromolecules, once the percolation network was formed. To gain more insight into the changes in the PLA chain relaxation processes, the obtained data were displayed in a Cole–Cole plot, which is usually used to describe the viscoelastic properties of materials showing a relaxation time distribution. As depicted in Figure 8, unfilled PLA showed a single arc associated with a relaxation process characterized by a relaxation time distribution. The introduction of MWCNTs caused the appearance of a tail in the high viscosity region, which is indicative of the long-term relaxation of the restrained PLA chains. As the content of nanotubes exceeded the percolation threshold, the local relaxation arc disappeared, hence demonstrating that the relaxation of the whole nanocomposite is governed by the long-term relaxation of restrained macromolecules.

Pötschke and co-workers [101] documented a peculiar rheological response for poly(caprolactone) (PCL)/MWCNTs nanocomposites obtained by melt-mixing at different processing conditions. In particular, nanocomposites produced using different mixing speeds were characterized through dynamic oscillatory measurements, exhibiting a rheological response strongly affected by the shear stresses that the polymer experienced during processing. At the lowest selected mixing speed, nanocomposites showed a rheological response quite similar to that of unfilled PCL, due to the large size of the MWCNT agglomerates present in the material; as the mixing speed was increased, a pronounced increase in the complex viscosity and storage modulus was observed, especially in the low frequency region, as a result of the improved dispersion of the nanofillers within the matrix. A further increase in the mixing speed caused a decrease in both rheological functions, despite the lower measured area of the nanofiller agglomerates. This finding was ascribed to a shortening of MWCNTs as a result of the high shear stresses developed at high mixing speeds, causing a decrease in the nanofiller aspect ratio and a consequent reduction in the ability of nanofillers to form a percolated network affecting the relaxation dynamics of PCL chains.

Vega et al. [102] evaluated the rheological behavior of PCL-based nanocomposites containing pristine MWCNTs and nanohybrids obtained by grafting low-molecular-weight PCL chains on nanofillers. All the produced systems exhibited a solid-like behavior, involving the appearance of a yield stress even at the minimum nanofiller content, due to the presence of a 3D percolated network of nanofillers in the melt; the formation of this network was further confirmed through the application of the percolation theory. Interestingly, nanocomposites containing low loadings of PCL-grafted nanofillers exhibited lower complex viscosities and storage moduli in oscillatory shear and higher compliance in creep, as compared to the unfilled matrix; this behavior, which disappeared for MWCNT loadings beyond 1 wt.%, was associated with competition between two opposite effects: a plasticization action exerted by the low-molecular-weight PCL chains grafted to nanotubes, which is prominent at low nanofiller amounts, and the effect of the formed nanofiller network, governing the overall rheological response at higher loadings.

## 3. Rheological Behavior of Polymer Blends Filled with CNTs

Generally, due to their low mixing entropy, polymeric materials are intrinsically immiscible, presenting a typical drop-matrix microstructure that often prevents the obtainment of superior properties. In fact, the final performance of a polymer blend can be optimized if the non-spherical domains formed in the melt state upon the shear flow field that the polymers experienced during processing are preserved; in this way, it is possible to achieve co-continuous morphologies, in which the mutual interpenetration of the two phases results in a synergistic combination of the properties of the components [103]. A common strategy exploited to manipulate the final microstructure of polymer blends involves the introduction of nanoparticles (such as CNTs), able to stabilize the polymer/polymer interface and to suppress the phase coarsening, thus promoting the obtainment of stable co-continuous morphologies [104,105]. When nanofillers are mixed with polymer blends, their localization in one of the polymer phases or in the interfacial region depends on different factors, including both thermodynamic and kinetic parameters [106]; the evaluation of the rheological response of filled polymer blends allows the prediction of the preferential localization of embedded nanofillers and the tuning of the final blend microstructure according to the desired final properties of the material [107].

The main achievements regarding the rheological behavior of polymer blends containing CNTs are listed in Table 1; in general, notwithstanding the preferential localization of embedded nanofillers, the introduction of nanotubes caused an increase in the viscosity and dynamic modulus values, as compared to those in the unfilled blend, with the appearance of a solid-like behavior resulting from the formation of a percolative network. Furthermore, some peculiar rheological responses were reported for systems, in which migration phenomena for CNTs occurred during annealing treatments. 

In this context, Hwang et al. [121] subjected to a thermal treatment PP/PS blends loaded with different amounts of MWCNTs, aiming to verify the effects of nanofillers on the coalescence phenomena usually observed for thermodynamically unstable polymer blends at high temperatures. The time evolution of the storage modulus showed a rapid decrease in this viscoelastic property for the unfilled PP/PS blend, although the single components exhibited long-term stability; this behavior was associated with the occurrence of phase coarsening, inducing an increase in the dimension of the dispersed phase domains, and a consequent decrease in the storage modulus of the blend. Conversely, PP/PS/MWCNT systems exhibited an increasing trend for the storage modulus as a function of the annealing time, indicating that the presence of nanotubes effectively prevented coalescence; further measurements performed after the thermal treatment demonstrated the rebuilding of the CNT network structure, through the Brownian motion of the nanofillers into the melt. A similar behavior was disclosed by Chen and co-workers [122] for PP/PMMA/MWCNT ternary systems subjected to a thermal treatment at 200 °C after processing. Additionally, in this case, a decrease in the storage modulus of the unfilled blend was observed with an increase in the annealing time, due to the progressive reduction of the interfacial area as a result of the coarsening and coalescence of the dispersed phase. The introduction of CNTs gave rise to a different behavior, involving an increase in the modulus over time, especially at the early stages of the treatment; furthermore, the observed increase in the modulus was found to be very sensitive to the nanotube content, suggesting that the restriction of the motion of macromolecular chains preventing the coalescence is progressively more pronounced as the MWCNT loading is increased.

**Figure 9:**
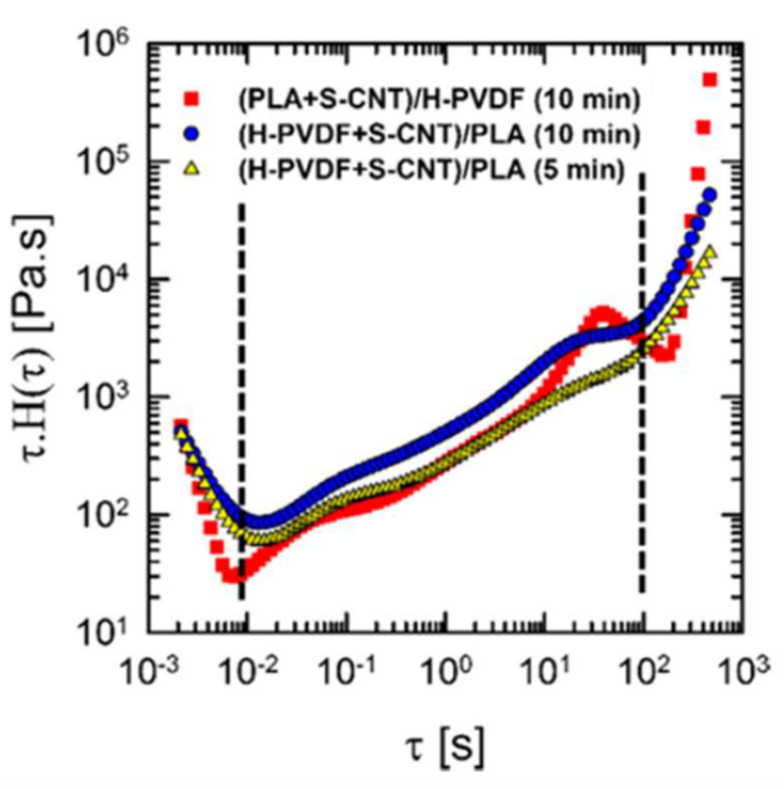
Weighted relaxation spectra of PLA/ Polyvinylidene fluoride (PVDF)/CNT systems ((PLA + S-CNT)/H-PVDF: premixed PLA/S-CNT + PVDF; (H-PVDF + S-CNT)/PLA: premixed H-PVDF/S-CNT + PLA)). Reprinted with permission from [123]. Copyright (2019) American Chemical Society.

Salehiyan and co-workers [123] evaluated the effects of the processing time and method on the viscoelastic response of PLA/ Polyvinylidene fluoride (PVDF)/MWCNT composites, using rheological characterization to assess the preferential localization of nanofillers. More specifically, the weighted relaxation spectra as a function of the frequency for blends containing 0.25 wt.% of nanofillers prepared through different protocols were derived (Figure 9), revealing a remarkable influence of the mixing sequence on the CNT localization and, consequently, on the relaxation dynamics of the polymer chains. When MWCNTs were premixed with PLA, they remained preferentially located in the PLA major phase: the low viscosity of the latter allowed the formation of interconnected CNT structures. This feature was supported by the appearance of a relaxation peak at τ = 39 s. Conversely, when nanotubes were introduced in the dispersed PVDF phase, the relaxation peak disappeared, suggesting an incomplete relaxation of PVDF/MWCNT domains; as the mixing time was increased, the appearance of a shoulder at about 23 s was observed, indicating some migration phenomena, with embedded nanofillers moving toward the interface and into the PLA phase.

## 4. Conclusions

In this review, the typical features of the rheological behavior of polymer-based nanocomposites containing carbon nanotubes were thoroughly discussed. In general, the assessment of rheological behavior represents a very effective tool for evaluating the internal microstructure of polymer/CNT complex systems, as the dispersion of the nanofillers and the interactions established between polymer chains and embedded nanostructures strongly affect the viscoelastic functions of the material. Furthermore, the knowledge of the material rheological response allows the gaining of a fundamental insight into the processability characteristics of the nanocomposites.

The incorporation of different CNT loadings within thermoplastics results in remarkable alterations of the macromolecule relaxation dynamics, giving rise to the following general modifications of the polymer’s rheological response:

- Higher values of viscosity and dynamic modulus are obtained in the resulting nanocomposites, as compared to those in the unfilled matrices, and, with an increase in the filler loading, the formation of an interconnected network structure is reached at a critical filler content known as the rheological percolation threshold, which strongly depends on the structural characteristics of the nanotubes.
- The formed interconnected network structure of anisometric fillers leads to an apparent yield stress, which is recognizable in a non-terminal behavior resulting in the appearance of a plateau in the modulus curves at low frequencies.
- Usually, CNTs have a modest effect on the high-frequency response and a significant influence on the low-frequency response, indicating that the embedded nanotubes are able to influence the polymer’s relaxation dynamics at length scales longer than the entanglement distance. This finding implies a minimal impact of the embedded nanofiller on the polymer processability.
- CNTs affect the non-linear viscoelastic responses of the polymer matrices, causing an anticipation of the transition toward the non-linear viscoelastic region.
- When CNTs are incorporated in a polymer blend, a different rheological behavior can be observed, mainly depending on the preferential localization of the embedded nanofiller.

Depending on the type of the polymer matrix, especially concerning its macromolecular architecture and chemical nature, CNT-containing nanocomposites exhibit peculiar rheological responses, reflecting the specific established polymer/CNT interactions that result in different arrangements of embedded nanofillers within the host polymer. The main achievements regarding the rheological behavior of CNT-containing systems based on different thermoplastic matrices are briefly highlighted in Table 2.

Finally, although a significant amount of experimental research is available in the literature on the rheological behavior of CNT-filled polymers, further theoretical and simulation studies would be very useful to fully understand the microstructure/processing/property relationships of these materials. Particular focus should be given to the assessment of the rheological response of CNT-containing nanocomposites under different flow fields commonly employed in the typical melt processing operations for thermoplastics: in fact, a detailed understanding of the viscoelastic behavior of CNT-based materials, strictly related to their microstructure, is essential for the successful development of CNT nanocomposite manufacturing and applications.

## Figures and Tables

**Table 1:** Main rheological results for CNT-containing polymer blends.

Blend Constituents	CNT Amount (wt.%)	Processing Method	Rheological Behavior	Ref.
Polypropylene/Polylactide	0.5–2	Melt-mixing	Solid-like behavior induced by nanofiller network	[108]
Polycarbonate/Polypropylene	1–10	Melt-mixing	Non-terminal rheological response at low frequencies	[109]
Polypropylene/Polyamide12	0.94–3.67	Melt-mixing and injection molding	Pseudo-plastic behavior	[110]
Polystyrene/Polylactide	2	Dilution of a masterbatch by melt-mixing	Mixed viscoelastic/solid-like due to the incomplete formation of the nanotube network	[111]
Polylactide/Polyethylene Oxide	1–2	Solution casting	A viscosity model was proposed, predicting complex modulus as a function of zero viscosity, yield stress, relaxation time and network breaking time	[112]
Polylactide/Polyethylene Oxide	0.29–2.91	Melt-mixing	Formation of a (pseudo-)network structure	[113]
Polylactide/Polybutylene Adipate Terephthalate	0.25–4	Melt-mixing	Appearance of a distinct shear-thinning behavior	[114]
Polylactide/Crosslinked Polyurethane	0.1–1	Melt-mixing	Transition from liquid-like to solid-like response	[115]
Styrene Acrylonitrile/Polyphenylene Ether	1	Solution casting and melt-mixing	Migration of functionalized CNTs into the Polyphentlene phase, leading to a decrease in both dynamic modulus and complex viscosity.	[116]
Polyethylene Oxide/Poly(methyl methacrylate)	0.5–4	Melt-mixing	Absence of zero-shear viscosity for CNT contents exceeding 4 wt.%	[117]
Polybutylene Succinate/High Density Polyethylene	0.29-2.91	Melt-mixing	Formation of a pseudo-network structure leading to enhanced complex viscosity and storage modulus	[118]
Polyamide6/Acrylonitrile Butadiene Styrene	0.05–0.25	Melt-mixing	Increased viscosity and storage modulus due to a reinforcing effect of the interface by the presence of MWNT	[119]
Polylactide/Polyethylene Oxide	1–2	Solution-mixing	Different values of the low frequency modulus were obtained depending on the prevailing effect between relaxation of Polyethylene oxide droplets and formation of CNT network	[120]

**Table 2:** Summary of the main achievements in the rheological behavior of CNT-containing thermoplastics.

Type of Matrix	Highlights
PC	■ Model matrix to inspect the effect of CNT introduction on the rheological behavior of polymer-based nanocomposites [45,46,47,48]. ■ The introduction of CNTs caused the appearance of a non-terminal behavior related to a liquid-to-solid transition due to a combined filler/filler–polymer/filler network [49]. ■ The utilization of low molar mass PC had a beneficial effect on the kinetics of network formation [55].
PE	■ A selective adsorption of the high molar mass fraction of PE chains onto the CNT surface was observed, causing a decrease in the entanglement density in the bulk material and a consequent lowering of the viscosity values in the composite systems [58,59,60]. ■ The Cox–Merz rule was not valid [59,61]. ■ In PEs with high molecular weights and broad molar mass distributions, the macromolecular architecture of the matrix was the main factor controlling the material rheological behavior, as the effects caused by the nanotube network were screened [62].
PP	■ Higher viscosity and modulus values as a function of CNT loading and a transition from liquid-like to solid-like behavior as the nanofiller content increases were observed [69,70,71,75,78]. ■ The formation of a percolative network was promoted by subjecting PP/CNT composites to a thermal treatment [72]. ■ PP matrices with high molar masses, able to develop high shear stresses during the flow, induced a preferential orientation of CNTs during the melt flow into a capillary, causing a decrease in the nanocomposite flow resistance and a consequent lowering of the material viscosity [74].
PS	■ In the case of functionalized CNTs, low amounts of nanofiller exerted a plasticizing action, reducing the matrix viscosity [85]. ■ In general, composites obtained through solution-mixing exhibited improved complex viscosities and lower percolation thresholds with respect to their melt-mixed counterparts, accounting for a general better dispersion of nanofillers and higher length of CNTs [89,90].
PA	■ A remarkable increase in complex viscosities and dynamic moduli, together with the disappearance of the terminal behavior, marking the transition from liquid-like to solid-like viscoelastic response was observed [92]. ■ The incorporation of CNTs resulted in a higher activation energy for the polymer flow process [93].
Biopolymers	■ A significant modification of the low-frequency relaxation of polymer chains, resulting from the interference of the CNT percolated network with the long-range motion of polymer macromolecules, was recorded [98,99,100]. ■ In the case of PCL-based nanocomposites, a remarkable influence of the processing conditions on the material rheological response was observed [101].
